# Genetic alterations in B cell lymphoma subtypes as potential biomarkers for noninvasive diagnosis, prognosis, therapy, and disease monitoring

**DOI:** 10.3906/biy-1908-23

**Published:** 2020-02-17

**Authors:** Esra ESMERAY, Can KÜÇÜK

**Affiliations:** 1 İzmir Biomedicine and Genome Center, İzmir Turkey; 2 İzmir International Biomedicine and Genome Institute, Dokuz Eylül University, İzmir Turkey; 3 Department of Medical Biology, Faculty of Medicine, Dokuz Eylül University, İzmir Turkey

**Keywords:** Hodgkin’s lymphoma, B cell non-Hodgkin’s lymphoma, genetic alterations

## Abstract

Neoplastic transformation of germinal center B (GCB) cells may give rise to a variety of different B cell lymphoma subtypes, most of which show substantial heterogeneity in terms of genetic alterations and clinical features. The mutations observed in cancer-related genes in GCB cells are related to abnormalities in the immunogenetic mechanisms associated with germinal center reaction. Recent studies have rapidly identified genomic alterations in B cell lymphomas that may be useful for better subclassification, noninvasive diagnosis, and prediction of response to therapy. The WHO recognizes different lymphoma subsets classified within 2 major categories of B cell lymphoma: Hodgkin’s lymphoma (HL) and B cell non-Hodgkin’s lymphoma (NHL), each with distinct genetic aberrations, including chromosomal translocations, copy number abnormalities, or point mutations. Next-generation sequencing-based technologies have allowed cancer researchers to identify somatic mutations and gene expression signatures at a rapid pace so that novel diagnostic or prognostic biomarkers, as well as therapeutic targets, can be discovered much faster than before. Indeed, deep sequencing studies have recently revealed that lymphoma-specific somatic mutations may be detected in cell-free circulating DNA obtained from the peripheral blood of B cell lymphoma patients, suggesting the possibility of minimally invasive diagnosis, monitoring, and predicting response to therapy of B cell lymphoma patients. In this study, the current status of the recurrent genetic aberrations observed during diagnosis and/or relapse in HL and the major subtypes of B cell NHL (i.e. diffuse large B cell lymphoma, follicular lymphoma, mantle cell lymphoma, and Burkitt lymphoma) are discussed to shed light on their potential use as noninvasive diagnostic or prognostic biomarkers and to reveal their role in lymphomagenesis as a target in therapy for newly diagnosed and chemotherapy-resistant cases.

## 1. Introduction

The human immune system uses complex defense mechanisms made up of cellular and humoral components that defend our body against a variety of pathogens. Our body is constantly protected against various microbial organisms with the help of a functional immune system. Lymphocytes are a group of white blood cells and critical cellular components of the immune system which protect us from pathogenic microorganisms and tumor formation. Lymphocytes fall into three main categories: B, T, and NK cells. B lymphocytes differentiate into plasma cells which secrete antibodies against specific antigens displayed on pathogens (Slifka et al., 1998). On the other hand, in addition to their immunoregulatory roles, T and NK cells are cytotoxic cells with the capacity to kill virus-infected or neoplastic cells by stimulating apoptosis (Bevan, 2004; Vivier et al., 2008). Proper functioning of the immune system depends on a fine balance between activation and termination of their activation in a timely manner; otherwise, dysregulated immune cell homeostasis may lead to uncontrolled growth of immunocytes. Somatic mutations of genes regulating cancer-associated biological processes including, but not limited to, cell cycle or apoptosis in lymphocytes may lead to uncontrolled cell growth, thereby resulting in the development of various types of lymphoma.

The majority of lymphomas (90%–95%) originate from B cells undergoing neoplastic transformation (Küppers, 2005); on the other hand, the cells of origin of the remaining lymphomas are either T or NK cells. B cell lymphomas are usually associated with defects in immunogenetic mechanisms operating as a part of the germinal center reaction, which plays a role in T cell dependent B cell activation and immunity (Küppers, 2005). Lymphoma is classified into two main groups: Hodgkin’s lymphoma (HL) and non-Hodgkin’s lymphoma (NHL). The first type of lymphoma described in history was HL, which was discovered by Thomas Hodgkin (van Gijn and Gijselhart, 2012). HL constitutes approximately 10% of all lymphomas (Küppers, 2005). The different types of lymphoma that were discovered after HL include other B cell lymphoma subtypes (e.g., diffuse large B cell lymphoma and follicular lymphoma), all of which are collectively much more frequent than subtypes of T or NK cell lymphomas. Subtypes of B cell lymphoma show substantial heterogeneity with respect to genetic and clinical characteristics. In the following sections, we discuss the somatic mutations involved in the formation of various subtypes of B cell lymphoma.

## 2. Genetic alterations identified in Hodgkin’s lymphoma 

**Figure 1:**
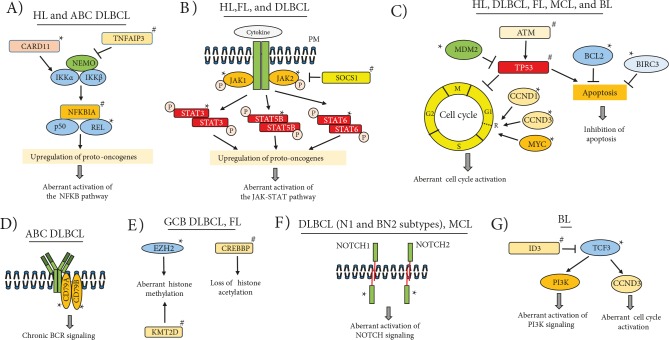
Mutated genes and affected pathways or biological processes in Hodgkin’s lymphoma and B cell non-Hodgkin’s lymphoma subtypes. HL: Hodgkin’s lymphoma; DLBCL: diffuse large B cell lymphoma; ABC: activated B cell type; GCB: germinal center B cell type; FL: follicular lymphoma; MCL: mantle cell lymphoma; BL: Burkitt lymphoma; *: gain of function mutations; #: loss-of-function mutations; P: phosphate; R: restriction point; PM: plasma membrane.

HL, first described by Thomas Hodgkin in 1832, is also known as Hodgkin’s disease (van Gijn and Gijselhart, 2012). The World Health Organization classifies HLs into two main categories: nodular lymphocyte predominant HL and classical Hodgkin’s lymphoma (cHL) (Swerdlow et al., 2016). cHL represents 90%–95% of HLs and has poorer prognosis compared to the other category (Shanbhag and Ambinder, 2018). It has four subtypes: 1) nodular sclerosis (NSCHL); 2) lymphocyte-rich (LRCHL); 3) mixed-cellularity (MCCHL), and 4) lymphocyte-depleted (LDCHL). HL develops with clonal proliferation of multinucleated, special type germinal center B cells called Reed–Sternberg cells (Kanzler et al., 1996). Hodgkin Reed–Sternberg (HRS) cells are tumor cells with no B cell characteristics and do not express the B cell receptor (BCR) on their surface (Farrell and Jarrett, 2011). Under normal conditions, these GCB cells are expected to undergo apoptosis in the absence of BCR signaling. This suggests that survival signals normally originating from active BCR signaling need to be sustained through alternative pathway(s) in HRS cells to compensate for the lack of BCR expression. In this context, HRS cells keep surviving due to deregulation of various signal transduction pathways and transcription factors, especially nuclear factor NF-κB (Figure 1A). *REL* locus amplifications are responsible for activation in roughly 50% of HL cases with constitutively active NF-κB signaling (Barth et al., 2003). In a significant proportion of cases, irregular activation of the NF-κB signaling pathway is believed to be related to loss-of-function mutations in tumor suppressor genes (e.g., *NFKBIA*, *TNFAIP3*) that inhibit the NF-κB signaling pathway (Weniger and Küppers, 2016).

Genetic analysis of cHL tumors is often difficult as tumor cellularity is less than 5% in biopsies. A recent study involving the microdissection method revealed mutated genes in the JAK-STAT signaling pathway (i.e. *STAT3*, *STAT5B*, *JAK1*, *JAK2*, and *PTPN1*) in 87% of cHL cases (Tiacci et al., 2018) (Figure 1B). Moreover, in a significant fraction of cHL cases, it has been reported that the JAK-STAT5 signaling pathway is activated due to mutations in SOCS1, an inhibitor of this pathway (Weniger et al., 2006). The *LMP2A* gene encoded by the Epstein-Barr virus (EBV) was shown to contribute to the development of EBV+ HLs through inducing transcriptional changes similar to those in HRS cells (Portis et al., 2003). Chromosomal translocations involving the *BCL6* oncogene were reported in approximately half of nodular lymphocyte predominant HL cases (Wlodarska et al., 2003). In the majority of HLs, gains of *MDM2 *or loss-of-function mutations of *TP53* have been reported, which suggests that the P53 signaling pathway is probably not functional in cases with these genetic aberrations (Küppers, 2009) (Figure 1C). Table 1 shows the recurrent genetic alterations and the dysregulated biological pathways in HL.

**Table 1:** The major genetic aberrations identified in Hodgkin’s lymphoma.

Lymphomatype	Gene	Genetic aberration	Frequency of mutated cases (%)	Dysregulated biological process or pathway	References
Hodgkin’s lymphoma	REL	Amplification	~50	NF-κB pathway	Barth et al., 2003
	NFKBIA, NFKBIE, TNFAIP3	Point mutations, deletions	50–60		Weniger et al., 2016
	MDM2	Gains	60	P53-dependent biological processes (e.g., cell cycle arrest and apoptosis)	Küppers, 2009
	TP53	Point mutations, deletions	10		
Classical Hodgkin’s lymphoma	JAK1, STAT3, STAT5B	Missense mutations	15	JAK-STAT pathway	Tiacci et al., 2018
	JAK2	Gains	32		
	PTPN1	Splice-acceptor, missense mutations	6		
	SOCS1	Frameshift mutations, disruptive in-frame deletions, splice donor, missense	47	JAK-STAT5pathway	Weniger et al., 2006;Tiacci et al., 2018
EBV+ Hodgkin’s lymphoma	LMP2A	EBV-encoded LMP2A mediated transcriptional changes	~50	Transcriptional signature similar to that of HRS* cells	Portis et al., 2003

*: Reed–Sternberg cells of Hodgkin’s lymphoma.

It has been known for a long time that HL cases respond to anti-CD30 antibody-drug conjugates, even in the presence of relapse (Falini et al., 1992; Schnell et al., 2002). However, until recently, the cell of origin of HRS cells in HL was not clear. A recent study by Weniger et al. showed that the transcriptomic profile of HRS cells is highly similar to that of CD30+ B cells, a rare B cell subset inside germinal centers (Weniger et al., 2018). In the same study, the transcriptional differences between the CD30+ B cell subset and HRS cells of HL were compared, and this showed significant downregulation of genomic stability regulators and cytokinesis. 

## 3. Genetic alterations in B cell non-Hodgkin’s lymphoma 

A great majority of NHL subtypes are B cell NHLs associated with abnormalities in immunogenetic mechanisms which operate in germinal center B cell reaction (Küppers, 2005). In this section, we address recurrent genetic alterations observed to date in different B cell NHL subtypes and oncogenic signaling pathway dysregulations associated with these alterations. 

### 3.1. Diffuse large B cell lymphoma

Diffuse large B cell lymphoma (DLBCL) is the most common type of NHL, accounting for 30%–40% of all cases (Dubois and Jardin, 2018). Based on gene expression profiling, DLBCL consists of two distinct subtypes, namely germinal center B cell (GCB) type and activated B cell (ABC) type (Alizadeh et al., 2000). This study is very critical in terms of the value of DNA microarray-based gene expression profiling for identification of new disease subtypes with prognostic differences, which cannot be identified with routine histological evaluations. Another study confirmed that DLBCL cases can be subclassified into two different molecular groups as GCB and non-GCB type with the tissue microarray (TMA) method and immunohistochemical analysis of CD10, BCL6, and MUM1 protein expression (Hans et al., 2004). Subsequent studies showed that these two DLBCL subtypes are genetically and biologically distinct. Uncontrolled, constitutive activation of the prosurvival NF-κB signaling pathway was observed in the ABC DLBCL subtype but not in GCB DLBCL cases (Davis et al., 2001). Indeed, *CARD11* mutations associated with activation of NF-kB were identified in approximately 10% of ABC DLBCL cases (Lenz et al., 2008) (Figure 1A). In ABC DLBCL cases, the NF-κB signaling pathway can continuously be activated by ‘chronic’ stimulation of the B cell receptor (BCR), and somatic mutations of two BCR subunits (i.e. *CD79A* and *CD79B*) may contribute to this oncogenic activation (Davis et al., 2010) (Figure 1D). In more than half of ABC DLBCL cases, deletions or inactivating mutations in the *TNFAIP3 *(A20) tumor suppressor gene and genetic defects in some other protooncogenes and tumor suppressor genes that regulate the NF-κB signaling pathway were found to have activated this signaling pathway irregularly (Compagno et al., 2009). Moreover, oncogenic *MYD88* mutations leading to constitutive activation of the JAK-STAT signaling pathway were observed very frequently in ABC DLBCL cases (Ngo et al., 2011). A very recent study by Jain et al. identified a novel protooncogene called *TCF4* (E2.2), which is located in the recurrently gained 18q21.2 locus in ABC DLBCL cases. Overexpression of TCF4 by Jain et al. showed direct transcriptional activation of *MYC* and *IGHM* genes in ABC DLBCL cell lines and cytotoxicity when it was knocked down (Jain et al., 2019). 

t(14;18)(q32;q21) translocation that increases BCL2 expression was detected in 20% of DLBCL cases (Willis and Dyer, 2000). Importantly, point mutations and indels of BCL2 were reported in GCB type DLBCL cases (Schuetz et al., 2012). The *BCL6* protooncogene was observed to be deregulated due to t(3;14)(q27;q32) translocation in 5%–10% of DLBCL cases (Willis and Dyer, 2000). Additionally, somatic mutations in *BCL6* disrupting the negative autoregulation of its expression were reported in DLBCL cases (Pasqualucci et al., 2003). It is important to note that some of the most critical genetic alterations specifically detected in GCB DLBCL cases to date were the point mutations affecting the oncogenic histone methyltransferase EZH2 on the Tyr641 residue in 21.7% of GCB DLBCL cases (Morin et al., 2010) (Figure 1E). EZH2 Tyr641 gain-of-function mutations were shown to increase H3K27 trimethylation, and this chromatin modification may be responsible for the silencing of tumor suppressor genes in mutated DLBCL cases (Yap et al., 2011). The major genetic aberrations observed in DLBCL cases are shown in Table 2. 

**Table 2:** The major genetic aberrations observed in diffuse large B cell lymphoma.

Lymphoma type	Gene	Genetic aberration	Frequency of mutated cases (%)	Dysregulated biological process or pathway	References
DLBCL	BCL2	t(14;18)(q32;q21)	20	Intrinsic pathway of apoptosis	Willis et al., 2000
GCB DLBCL	BCL2	Point mutations, indels	43	Intrinsic pathway of apoptosis	Schuetz et al., 2012
DLBCL	BCL6	t(3;14)(q27;q32)	5–10	Germinal center B cell reaction	Willis et al., 2000
DLBCL	BCL6	Somatic point mutations	16	Disruption of negative autoregulation of BCL6 expression	Pascualucci et al., 2003
ABC DLBCL	CARD11	Missense mutations in coiled-coil domain	9.6	NF-κB pathway	Lenz et al., 2008
	CD79B	Y196 ITAM mutation, ITAM deletion	21	Chronic BCR signaling, NF-κB pathway	Davis et al., 2010;Schmitz et al., 2018
	CD79A	ITAM deletion, splice site mutation	2.9		
GCB DLBCL	EZH2	Point mutations on Tyr641	21.7	Trimethylation of Lys27of histone H3 (H3K27)	Morin et al., 2010
DLBCL	MYC	t(8;14)(q24;q32)	10	G1 phase of the cell cycle	Willis et al., 2000
ABC DLBCL	MYD88	Missense mutations	37	JAK-STAT pathway	Ngo et al., 2011;Schmitz et al., 2018
DLBCL (N1 andBN2 subtypes)	NOTCH1, NOTCH2	Frameshift truncating mutations	11.7	Notch signaling pathway	Arcaini et al., 2015
ABC DLBCL	TNFAIP3 (A20)	Nonsense mutations, frameshift indels, splice site mutations, deletions	~55	NF-κB pathway	Compagno et al., 2009
ABC DLBCL	TCF4	Gain/amplification	40.7	Transcriptional activatorof IGHM and MYC	Jain et al., 2019

ABC: Activated B cell type; GCB: germinal center B cell type; ITAM: immunoreceptor tyrosine-based activation motif; BCR: B cell receptor.

As a result of the above-mentioned studies that reveal differences in genomic, transcriptomic, biological, and clinical characteristics in ABC and GCB type DLBCL cases, the WHO provisionally defined the ABC and GCB types as 2 distinct subclasses of DLBCL. However, some other DLBCL subtypes were also recognized by WHO based on other characteristics such as presence of chronic inflammation or viral infection in primary tumors (Swerdlow et al., 2016). Although the molecular classification dividing DLBCL into the ABC and GCB subgroups is generally accepted, 10%–20% of cases cannot be classified using this method (Dubois and Jardin, 2018). While discussions on DLBCL subtypes persist, a quaternary molecular classification system for DLBCL has recently been proposed based on results of extensive genomic and transcriptomic analyses. Subtype nomenclature proposed for this classification is as follows: MCD for *MYD88*, *L265P*, and *CD79B* mutated cases; BN2 for *BCL6* fusion genes or *NOTCH2* mutated cases; N1 for *NOTCH1* mutated cases; and EZB for *EZH2* mutated or BCL2 translocated cases (Schmitz et al., 2018) (Figure 1F). This newly proposed classification method stemming from various oncogenic mutations frequently observed in DLBCL cases aims to ensure selection of more appropriate treatment approaches for genetically and clinically heterogeneous DLBCL cases, thereby improving ABC and GCB classification instead of replacing these two subtypes. 

### 3.2. Follicular lymphoma 

Follicular lymphoma (FL) is the most common type of indolent lymphoma, and it is the second most frequent lymphoma among all NHLs after DLBCL (Shankland et al., 2012). Like other B cell NHL subtypes, development of FL is thought to be associated with irregularities in somatic hypermutation and class switch recombination mechanisms functional during GCB reaction. The most characteristic genetic alteration of FL is the t(14; 18) (q32; q21) IGH/BCL2 translocation that brings the active enhancer of the immunoglobulin heavy chain gene (IGH) closer to the *BCL2* gene (Tsujimoto et al., 1984). This translocation contributes to FL development through inhibition of apoptosis due to irregular, high expression of the antiapoptotic *BCL2* gene product (Capaccioli et al., 1996). t(14; 18) (q32; q21) translocation is observed in 70% to 95% of FL cases, and is used as a diagnostic biomarker of FL that can be detected by routine cytogenetic analyses (Einerson et al., 2005). Recent genomic analyses of FL cases have revealed mutations related to lymphomagenesis in genes of the JAK-STAT and NF-κB signaling pathways as well as in genes involved in posttranslational modification of histones such as *CREBBP*, *EZH2*, and *KMT2D* (*MLL2*) (Okosun et al., 2014) (Figure 1E). More than 50% of FL cases may show histological transformation to more aggressive cancers such as DLBCL and Burkitt lymphoma (Kridel et al., 2012). The life expectancy in cases of transformed FL (tFL) is as short as 1.7 years (Al-Tourah et al., 2008); therefore, ongoing investigations focus on identification of genetic alterations associated with histological transformation. Somatic point mutations of *BCL2* (Matolcsy et al., 1996; Correia et al., 2015), *TP53* (Lo Coco et al., 1993), *miR-142*, and *MEF2B* (Bouska et al., 2017) genes were reported to be associated with this transformation. Table 3 lists the recurrent genetic alterations observed in FL and/or tFL cases.

**Table 3:** Recurrent genetic alterations identified in follicular lymphoma, Burkitt lymphoma, and mantle cell lymphoma.

Lymphomatype	Gene	Genetic aberration	Frequency of mutated cases (%)	Dysregulated biological process or pathway	References
Follicular and/or transformed follicular lymphoma	BCL2	t(14;18)(q32;q21)	~80	Intrinsic pathway of apoptosis	Tsujimoto et al., 1984; Willis et al., 2000
	CREBBP	Somatic nonsynonymous mutations	64	Histone acetylation patterns	Okosun et al.,2014
	EZH2		20	H3K27me3 repressive marks	
	KMT2D (MLL2)		81	Histone lysine methylation patterns	
	SOCS1		8	JAK-STAT signaling	
	STAT6		12		
	MEF2B		20	B cell transcription factor	Bouska et al., 2017
	PAPOLG	Gain/amplification	24 in FL, 30 in tFL	Polyadenylation of transcripts	Kurşun et al., 2019
	REL		24 in FL, 30 in tFL	DNA damage inducedNFκB pathway	Hu et al., 2017
	MYC		15.7 in FL, 29.1 in tFL	Cell cycle and other oncogenic pathways	Bouska et al.,2014
	TP53	Deletion	12 in FL, 22 in tFL	P53 dependent cell cycle arrest and apoptosis	
	TNFAIP3			NF-κB pathway	
Burkittlymphoma	CCND3	Missense, nonsense,indel mutations	14.6	G1-S cell cycle transition	Schmitz et al.,2012
	ID3	Missense, nonsense, cyrptic splice site mutations	58.5	Pro-survival PI3 pathway, cell cycle	
	TCF3(E2A)	Missense mutations	29.2	Pro-survival PI3 pathway, cell cycle	
	MYC	t(8;14)(q24;q32)	100	c-MYC target genes involved in cell cycle regulation, metabolism etc.	Willis et al., 2000
Mantle cell lymphoma	ATM	Deletion, deleteriouspoint mutations	75	DNA damage response	Schaffner et al.,2000
	CCND1	t(11;14)(q13; q32)	95	G1-S cell cycle transition	Willis et al., 2000
	P53	Missense mutations	15	P53 dependent cell cycle arrest and apoptosis	Greiner et al.,1996
	NOTCH1	Truncating mutations, frameshifting indels	12	NOTCH signaling pathway	Kridel et al., 2011
	BIRC3	Deletion, splice site mutations	10	Apoptosis	Beà et al., 2013
	MEF2B	Missense mutations (p.K23R and p.N49S)	7	Unknown	
	NOTCH2	Truncating mutations	5	NOTCH signaling pathway	
	WHSC1	Missense mutations (p.E1099K and p.T1150A)	14	Altered methylation of H3K36	

A recent article published by Andor et al. analyzed the transcriptome of 34,188 cells from 6 primary FL tumors to better understand the complexity of FL tumor biology for identification of novel, more effective targeted therapeutic approaches (Andor et al., 2019). In malignant B cell clones, the authors observed overexpression of *BCL2* and downregulation of *CD52*, *FCER2*, and MHC class II genes compared with the expression in normal B cells from the same patients. In the same single-cell RNA-Seq study, the authors also focused on expression profiles in tumor-infiltrating T cells, which showed genes simultaneously expressed with immune checkpoint molecules in regulatory T cells. This single cell-based study showed different subclones, implying intratumoral genetic heterogeneity of primary FL tumor tissues. 

### 3.3. Burkitt lymphoma 

Burkitt lymphoma (BL) is an aggressive type of pediatric and adult B cell lymphoma originating from GCB cells (Deffenbacher et al., 2012). Pediatric BL is the most common type of childhood B cell NHL, accounting for 80% of all childhood B cell NHLs (Minard-Colin et al., 2015). BL is clinically classified into three subtypes: sporadic, endemic, and immunodeficiency-related (Piccaluga et al., 2011). The most characteristic genetic aberration associated with BL cases is the juxtaposition of the IgH enhancer and *MYC* protooncogene due to t(8; 14) translocation, which leads to high and constitutive expression of MYC (Joos et al., 1992). MYC-induced apoptosis in BL cells is not functional in one-third of BL cases due to deletions or point mutations of P53 (Gaidano et al., 1991). For P53 WT BL cases, *MDM4*, which is located in the recurrently gained 1q locus, has recently been found to be overexpressed and to inhibit MYC-induced apoptosis through inhibition of P53 (Hüllein et al., 2019).

Oncogenic *CCND3* mutations stabilizing CCND3 protein isoforms and thereby disrupting cell cycle progression were identified in 38% of sporadic BL (sBL) cases (Schmitz et al., 2012). In the same study, mutations in the *TCF3* (E2A) transcription factor or its negative regulator, *ID3*, were reported in 70% of sBL cases (Figure 1G). It was proposed that mutated *TCF3* contributes to BL formation by stimulating survival through irregular activation of the phosphatidyl inositol-3-OH kinase (PI3K) signaling pathway (Schmitz et al., 2012). Another whole-genome and whole-exome sequencing-based study reported mutations in SWI/SNF family genes involved in chromatin remodeling such as *ARID1A* and *SMARCA4*, as well as those in *CCT6B*, *SALL3*, *FTCD*, and *PC* genes (Love et al., 2012). The major genetic aberrations and the biological processes affected by these alterations in BL cases are shown in Table 3.

One of the most critical characteristic features of BL is the presence of EBV in the genome of most BL tumors, especially in the endemic subtype (Shannon-Lowe et al., 2017). Integrative analyses of whole-genome and transcriptomic profiles have recently shown that EBV-positive tumors show a robust increase in abnormal somatic hypermutation in pediatric endemic and sporadic BL cases (Grande et al., 2019). This study has also shown genetic alterations in genes affecting BCR/PI3K signaling, MYC regulation, apoptosis, the SWI/SNF complex, and G-protein coupled receptor (GPCR) signaling. Another study also showed inactivating mutations in components of the GPCR signaling pathway (i.e. *GNA13* and *RHOA*) genes, suggesting that this pathway may be a target of therapy for GNA13 or RHOA-mutated BL cases (O’Hayre et al., 2016). 

### 3.4. Mantle cell lymphoma 

Mantle cell lymphoma (MCL) is a type of lymphoid cancer originating from neoplastic transformation of naive B cells that do not encounter antigens (Cortelazzo et al., 2012). MCLs are highly heterogeneous with respect to their morphological, clinical, and pathological characteristics. One of the most characteristic genetic alterations of MCL is the t(11; 14)(q13; q32) chromosomal translocation, which increases CCND1 expression that stimulates the cell cycle (Cortelazzo et al., 2012) (Figure 1C). Interestingly, highly proliferative MCL tumors express shorter but more stable versions of CCND1, generated through genomic deletions or point mutations ending up with shorter 3’ untranslated regions, which in turn results in poor survival of MCL patients (Wiestner et al., 2007). A recent study by Mohanty et al. investigated the consequence of CCND1 coding sequence point mutations (i.e. E36K, Y44D, or C47S) and they observed improved stability of CCND1 protein in these mutated MCL cells (Mohanty et al., 2016).

MCL can be clinically indolent or aggressive; the 5-year overall survival rate is approximately 60% in the low-risk group, whereas it decreases significantly in moderate or high-risk groups (Hoster et al., 2008). MCL was proposed to develop in 2 distinct ways. Classical MCL usually consists of SOX11+ cases with no *IGHV* gene mutations. On the other hand, other MCLs derive from cells with *IGHV *mutation but not *SOX11 *expression (Swerdlow et al., 2016). 

Previous studies have shown that deletions and inactivating mutations in the tumor suppressor *ATM* (Schaffner et al., 2000) or *TP53* (Greiner et al., 1996) may play a role in MCL development (Figure 1C). The impact of inactivating mutations of P53 may not be limited with MCL lymphomagenesis. Eskelund et al. reported that P53 mutation status is an important factor for increasingly poor prognosis of younger MCL patients, for which P53 mutations confer resistance to rituximab or autologous stem cell transplantation (Eskelund et al., 2017). Whole-transcriptome analyses of MCL tumor samples and cell lines showed *NOTCH1* mutations in 12% of MCL cases (Kridel et al., 2012). In another NGS-based study, cancer-associated mutations were observed in antiapoptotic *BIRC3*, Toll-like receptor 2 (*TLR2*), chromatin modification genes (*WHSC1*, *MLL2*, and *MEF2B*), and the *NOTCH2* gene (Beà et al., 2013). Table 3 shows the genetic alterations associated with the pathogenesis of MCL. 

## 4. Diagnostic, prognostic, and therapeutic implications of genetic alterations

Several studies revealed a clinical relevance for the identified genetic alterations in different subtypes of B cell NHL. These clinically relevant genetic alterations may be potentially useful in routine diagnosis as well as in risk stratification during diagnosis. The oncogenic chromosomal translocations originating from aberrantly operating class-switch recombination mechanisms are useful for genetic diagnosis of B cell NHL subtypes (Lenz et al., 2007). The presence of these chromosomal translocations that are specific for certain subtypes of B cell NHL can be used to evaluate clonality and to facilitate the diagnosis of the specific subtype of lymphoma. Interphase FISH is routinely used for detection of t(14;18) translocation (i.e. IGH-BCL2) in FL (Vaandrager et al., 2000), t(11;14) translocation (i.e. IGH-CCND1) in MCL (Monteil et al., 1996), or t(8;14) translocation (i.e. IgH-MYC) in BL (Siebert et al., 1998) for diagnostic purposes. 

Some of the identified genetic alterations may be useful to predict patient survival. It has been known for some time that *P53* mutations are associated with poor patient survival in aggressive B cell lymphomas (Ichikawa et al., 1997). Several studies have established a relationship between *P53* mutations and poor prognosis for different B cell lymphoma subtypes. As an example, Zainuddin et al. reported an inferior survival for GCB DLBCL cases with *P53* mutations (Zainuddin et al., 2009). Another report showed that *P53*-mutated MCL cases have a worse survival rate than that of unmutated ones (Halldórsdóttir et al., 2011). In support of these previous reports, a recent study based on metaanalyses showed a general prognostic significance for *P53* mutations in B cell NHLs (Xu et al., 2017). MYC overexpression due to genetic aberrancies (e.g., translocation and amplification) can be used as a prognostic biomarker for certain B cell NHL subtypes. Based on a recent report, *MYC* amplifications or rearrangements can independently predict overall survival in patients of DLBCL (Quesada et al., 2017). Application of next-generation sequencing (NGS)-based technologies such as whole exome sequencing revealed the genomic mutational landscape of different types of B cell lymphomas. Detection of these mutations may provide useful information regarding prognosis or prediction of response to immunochemotherapy. In one of these NGS-based studies, FFPE tumor samples of DLBCL patients were screened for the presence of recurrently identified mutations using high throughput sequencing (Juskevicius et al., 2017). After that, a relationship was established between the identified mutations and overall progression-free and event-free survival in R-CHOP-treated DLBCL patients, which showed poorer prognosis for patients with *CREBBP* and *EP300* mutations and a better prognosis for patients with *SOCS1* mutations. 

Somatic mutation status may add value to the traditionally used prognostic indexes in terms of better risk stratification of B cell NHLs. To address this possibility, Pastore et al. retrospectively analyzed the mutational status of tumor samples of 151 follicular lymphoma cases, which were obtained before patients received the first-line immunochemotherapy (Pastore et al., 2015). This report showed that evaluation of the mutation status of seven cancer-associated genes (i.e. *EZH2*, *ARID1A*, *MEF2B*, *EP300*, *FOXO1*, *CREBBP*, and *CARD11*) with deep sequencing improved prognostication of follicular lymphoma patients receiving first-line immunochemotherapy when integrated into FILIP1 and ECOG prognostic indexes, which in turn outperformed prognostications performed with FILIPI alone or FILIPI combined with ECOG performance status. 

Genetic alterations and transcriptional signatures may be used together or separately as biomarkers for improved diagnosis or prognosis of B cell lymphoma patients. Whether mutations or transcriptional signatures should be chosen as biomarkers depends on the potential of these genetic or transcriptional changes to separate B cell lymphoma subtypes into more distinct and refined diagnostic and/or prognostic subgroups. Subclassification of DLBCL cases into ABC and GCB subgroups using gene expression profiling may be one of the best examples not only in terms of improved cancer diagnosis and prognosis but also for identification of genetic alterations specific for each subtype. Lenz et al. showed that there are 30 genetic alterations observed in a DLBCL subtype specific manner (Lenz et al., 2008). For instance, *INK4a/ARF* tumor suppressor loci deletions were identified mainly in the ABC DLBCL subtype. On the other hand, *PTEN* deletions were observed in the GCB but not in the ABC subtype of DLBCL (Lenz et al., 2008). Overall, these observations suggest that using transcriptomic signatures for DLBCL subclassification during diagnosis may be superior to other classifications including genetic alterations. However, MYC/BCL2 coexpression, which is observed more commonly in the ABC subtype, was proposed as an independent prognostic factor regardless of the ABC versus GCB subtype classification (Hu et al., 2013). DLBCL cases with MYC/BCL2 coexpression showed remarkably poorer survival rates when compared to the rest of the cases. It should be noted that a distinct oncogenic gene expression signature was identified for the MYC/BCL2 coexpression group in this study. A transcriptional signature may be more useful than mutations for subdividing MCL cases into prognostically distinct diagnostic subgroups. A thirteen-gene signature including *SOX11* was identified to clearly distinguish indolent and conventional subtypes of MCL (i.e. iMCL and cMCL, respectively). SOX11 protein expression status in MCL cases may be useful to distinguish indolent and conventional subtypes of MCL, which differ in clinicopathological characteristics, including patient survival (Fernàndez et al., 2010). 

Identification of the aberrant signaling pathways in the subtypes of B cell lymphoma provides opportunities for development of targeted therapeutics with fewer side effects in comparison to traditional chemotherapeutic approaches. Recent clinical studies have focused on inhibition of oncogenic pathways for better treatment of B cell lymphoma patients. JAK-STAT pathway activation is frequently observed in cHL cases. Consequently, JAK1/2 inhibitors are already in clinical trials for treatment of advanced relapsed/refractory HL (Den Neste et al., 2018). A selective BCL2 inhibitor (venetoclax) was evaluated in 106 patients with relapsed or refractory B cell NHL (i.e. FL, DLBCL, and MCL) as a single agent for its activity and safety profile; however, future studies are needed maximize the therapeutic benefits for these patients (Davids et al., 2017). Genetic alterations in epigenetic regulatory genes offer significant therapeutic opportunities for treatment of FL and DLBCL patients with relapsed or refractory disease. To address this possibility, Italiano et al. recently investigated an EZH2 inhibitor (i.e. tazemetostat) in relapsed and refractory B cell NHL patients (Italiano et al., 2018). Based on these results, it was concluded that tazemetostat has a favorable safety profile and antitumor activity in patients with refractory B cell NHL. One of the most intriguing results regarding targeted therapeutics against a constitutively activated oncogenic pathway is selective inhibition of the chronic BCR signaling with ibrutinib in DLBCL. Consistent with the chronic activation of BCR signaling selectively in the ABC DLBCL subtype, ABC DLBCL patients responded significantly better to this BCR signaling inhibitor in comparison to patients with the GCB DLBCL subtype (Wilson et al., 2015). These and other similar studies show that genetic alterations that activate oncogenic signaling pathways may be pharmacologically targeted to improve currently available therapeutic options such that B cell lymphoma patients in advanced stages may benefit from more effective and safer treatment alternatives. 

## 5. Chemotherapy resistance-associated genetic aberrations in B cell NHL

One of the most important challenges in the treatment of B cell lymphoma patients is the resistance that develops against chemotherapy or immunochemotherapy treatment. After inclusion of rituximab in the traditional CHOP (cyclophosphamide, doxorubicin, vincristine, and prednisone) chemotherapy, CHOP therapy resistance was eliminated in many B cell lymphoma cases. Mounier et al. reported that R-CHOP therapy overcomes therapy resistance associated with BCL2 overexpression in elderly DLBCL patients (Mounier et al., 2003). A report by Liu et al. showed that PRDM1β is expressed in a subset of non-GCB-like DLBCL cases, and its expression is associated with poor survival in CHOP-treated but not R-CHOP-treated DLBCL cases. They further showed that rituximab downregulates PRDM1β expression in vitro (Liu et al., 2007). However, DLBCL cases may develop resistance to R-CHOP therapy. Yuan et al. showed that high microRNA-125b and microRNA-130a levels in the serum of DLBCL cases are associated with R-CHOP resistance in DLBCL cases (Yuan et al., 2016). Laursen et al. showed that high expression of CXCR4 on the tumor cell surface may be responsible for R-CHOP resistance in a subset of DLBCL cases (Laursen et al., 2019). Another study by Wilson et al. asked whether ABC DLBCL patients who have chemotherapy refractory disease can be treated through inhibition of BCR signaling. They performed a clinical trial with ibrutinib and observed a high response in those patients, especially in the presence of *MYD88* mutations (Wilson et al., 2015).

Follicular lymphoma cases may transform into high-grade lymphomas called transformed follicular lymphomas (tFL) such as DLBCL or BL, which are often chemotherapy-resistant (Lossos and Gascoyne, 2011). Many genetic alterations in FL tumors such as deletions of *P53*, *P16/CDK2NA*, or deregulations of *MYC* have been associated with FL to tFL transformation (Lossos and Gascoyne, 2011). Given the increased chemoresistance after histological transformation, these genetic alterations may also be responsible for chemotherapy resistance. Based on genome-wide copy number analyses of 198 FL and 79 tFL cases using SNP arrays, Bouska et al. identified recurrent chromosomal gains or losses in FL cases that can be potentially associated with tFL transformation. These chromosomal gains and losses may restrict immune surveillance and deregulate P53 or NFκB pathways (Bouska et al., 2014). *REL* is one of the protooncogenes identified to reside on the frequently amplified 2p16.1-p15 locus in FL and tFL cases based on this study. Recently, Hu et al. showed that FL cases with high REL expression levels may be associated with chemotherapy resistance due to activation of DNA damage-induced repair and cell cycle arrest pathways; however, future studies are needed to investigate whether REL is a predictive biomarker of chemotherapy in FL and/or tFL cases (Hu et al., 2017). Another recent study systematically analyzed genes in the frequently amplified 2p16.1-p15 locus by integrating an SNP array and gene expression profiling data of previously published reports on FL and tFL cases to address whether there are genes other than *REL* that may act as protooncogenes during transformation to tFL (Kurşun and Küçük, 2019). This study showed that *PAPOLG* is a candidate protooncogene for FL development, and it may have a role during histological transformation to tFL.

Current efforts in MCL genomics focus on identification of mutations associated with chemotherapy or immunochemotherapy resistance. To identify genetic alterations associated with therapy resistance in MCL cases, Wu et al. performed genome-wide analyses of MCL tumors which revealed cancer-related mutations at the time of diagnosis, as well as relapse (Wu et al., 2016). They identified recurrent *CARD11* mutations in 5.5% of MCL cases. More importantly, overexpression of mutated *CARD11* constructs in MCL cell lines conferred resistance to ibrutinib and lenalidomide, which are inhibitors of BCR and NF-κB signaling, respectively. Similarly, *CCND1* mutations described in MCL cases by Mohanty et al. conferred therapeutic resistance to ibrutinib, a bruton tyrosine kinase inhibitor used for MCL treatment (Mohanty et al., 2016). Agarwal et al. focused on the genomic profiles of MCL patients who respond or did not respond to ibrutinib and venetoclax combinatory therapy and identified a distinct genomic profile separating responders from nonresponders. Based on this very recent study, chemotherapy-resistant patients harbor 9p21.1–p24.3 locus deletion and/or mutations of the genes forming the SWI–SNF chromatin-remodeling complex (Agarwal et al., 2019). They observed that defective SWI–SNF complexes promote transcriptional upregulation of BCL-XL, which may confer resistance to the ibrutinib and venetoclax combination. This study is a good example of precision medicine and shows that individual patients’ tumor genotypes can be used to decide on the most appropriate therapy.

## 6. Noninvasive clinical applications of genomic alterations of B cell lymphoma for diagnosis, prognosis, or disease monitoring 

Traditional diagnosis of B cell lymphoma subtypes requires immunohistochemical and histopathological analyses of tumor biopsy sections. Tumor biopsies are obtained from lymphoma patients using invasive methodologies, which may have the following specific disadvantages: 1) procedures are generally time-consuming; 2) tissue sampling is localized; therefore, the obtained tissue may not reflect the genetic characteristics and heterogeneity of the entire tumor tissue present in the patient; 3) tumor biopsies may not always be easy to obtain; 4) patients may have some pain. Due to these disadvantages, cancer researchers have been trying to address whether body liquids (e.g., blood and saliva) contain tumor-derived biomolecules that reflect the genetic characteristics of solid tumor tissues of cancer patients. Circulating cell-free DNA (cfDNA) has proven not only to be useful in terms of overcoming the disadvantages of standard biopsy listed above but has also provided the opportunity to track genetic changes through multiple, periodical blood sampling and determine the emergence of therapy-resistant clones several months earlier than currently used methods in routine clinical settings (Crowley et al., 2013).

Several recently reported elegant studies have revealed the feasibility of cfDNA genotyping for diagnosis, prognosis, and monitoring chemotherapy response in major subtypes of B cell lymphoma. Spina et al. evaluated whether circulating cell-free tumor DNA can be used for cHL genotyping (Spina et al., 2018). By applying a custom-designed, ultradeep targeted NGS panel, which consisted of 77 genes recurrently mutated in B cell tumors, on DNA samples from 80 diagnosed and 32 refractory cHL cases, the authors observed that 87.5% of the somatic mutations detected in tumor biopsies were also present in the circulating cell-free tumor DNA (ctDNA) of the same patients’ plasma. The same study also revealed that decreased plasma ctDNA concentrations are associated with markedly better survival for cHL patients treated with a combination of chemotherapeutic drugs (i.e. adriamycin, bleomycin, vinblastine, and dacarbazine). Another recent study, by Rossi et al., showed that the sensitivity for detection of mutations in cfDNA is 83% if tumor tissue gDNA is regarded as the gold standard for mutation detection, suggesting that noninvasive blood sampling and subsequent plasma cfDNA genotyping may have diagnostic value (Rossi et al., 2017). The authors also observed the changes in variant allele frequency (VAF) before and after R-CHOP therapy, which showed that patients responding to R-CHOP have decreased VAF, whereas others did not. Importantly, new variants emerged in different patients such as those of *PIM1*, *BIRC3*, and* FBXW7.* This observation suggests that mutations responsible for relapse or refractory disease in individual DLBCL patients can potentially be determined noninvasively using a blood-based test. From a precision medicine perspective, cfDNA genotyping may assist clinicians in choosing the most suitable option for targeted therapy through facilitated detection of individual genetic subclones responsible for relapse in DLBCL patients. There are other recent studies that evaluated plasma cfDNA levels in DLBCL (Kurtz et al., 2018), addressing whether it can predict or monitor therapy response or if it can predict tumor burden or prognosis of FL cases (Delfau-Larue et al., 2018). Using droplet digital PCR, Alcaide et al. detected previously known mutations (e.g., EZH2 Y641 and STAT6 D419) in circulating tumor DNA in major B cell NHL subtypes (Alcaide et al., 2016). 

## 7. Conclusion

HL and B cell NHL, which represent most lymphoid cancers, include mutations related to malfunctioning immunogenetic mechanisms during germinal center reactions. These genetic aberrations may provide us with novel biomarkers of noninvasive diagnosis or prognosis if they distinguish B cell lymphoma subsets from similar B cell lymphomas or benign diseases and predict patient survival, respectively. Furthermore, biological signaling pathways dysregulated by activating mutations in B cell lymphomas offer new opportunities for targeted therapies.

## Acknowledgments

This study was supported by the Young Scientists Award Program of the Turkish Academy of Sciences (TÜBA GEBİP 2017). A part of this review was presented in Turkish in *Hematolog*, which is a members-only periodical magazine of the Turkish Society of Hematology.
